# Pollen limitation in a single year is not compensated by future reproduction

**DOI:** 10.1007/s00442-020-04623-x

**Published:** 2020-02-20

**Authors:** Matthew Tye, Johan P. Dahlgren, Nina Sletvold

**Affiliations:** 1 Department of Ecology and Genetics, Uppsala University, SE-752 36 Uppsala, Sweden; 2 Department of Biology and The SDU Interdisciplinary Centre on Population Dynamics, University of Southern Denmark, 5230 Odense, Denmark

**Keywords:** Cost of reproduction, Demographic compensation, Long-lived perennial, Pollen limitation, Orchidaceae

## Abstract

Seed production is critical to the persistence of most flowering plant populations, but may be strongly pollen limited. To what extent long-lived plants can compensate pollen limitation by increasing future reproduction is poorly understood. We tested for compensation in two *Dactylorhiza* species that differ in reproductive investment by experimentally reducing and increasing pollination in two independent annual cohorts and monitoring demographic responses in the subsequent 2 years for the 2014 cohort and in 1 year for the 2015 cohort. Demographic rates in the second year were significantly affected by pollination treatment in both species, but specific responses differed both between species and years. There was no effect of pollination treatment on demographic responses in the third year. In sum, effects were too weak to make up for the lost reproduction; total fruit production across all 3 years was by far highest in the increased pollination treatment in both species. These results show that long-lived plants do not necessarily compensate for pollen limitation by increasing future reproduction. It further suggests that even periodic declines in pollination rates may have severe demographic consequences, particularly in populations where germination is not density dependent. This has implications for predicting plant population viability in response to changes in pollination intensity.

**Electronic supplementary material:**

The online version of this article (10.1007/s00442-020-04623-x) contains supplementary material, which is available to authorized users.

## Introduction

Pollen availability can be a strong limiting factor of many plant species’ reproduction (Burd 1994; Larson and Barrett 2000; Ashman et al. 2004; Knight et al. 2005), and is expected to become an increasingly important issue in light of global decreases in overall abundance and condition of pollinators (Biesmeijer et al. 2006; Potts et al. 2010). In addition, increased climatic variability in some regions (e.g. Schär et al. 2004) may lead to higher variation in pollination success. In species whose population growth is limited by seed input, increasing strength and variability of pollen limitation may lead to population declines and local extinctions, particularly if selfing or clonal reproduction is absent (cf. Lennartsson 2002; Biesmeijer et al. 2006). It is thus crucial to understand how plant populations respond demographically to changes in pollination rates and, in particular, to years of low pollination success.

Perennial plants may respond in several ways to a year of poor reproductive output caused by pollen limitation. Individuals may lack demographic compensation in which they either have similar fecundity in the future regardless of pollination rate, or do not increase reproduction in subsequent years sufficiently to make up for the lost reproduction. Alternatively, individuals may increase reproduction to compensate for the lost reproductive opportunity, or increase future reproduction to more than make up for the loss (i.e., overcompensation) by increasing fecundity per reproductive bout over subsequent years (Easterling et al. 2000; Samson and Werk 1986). Individuals may also redirect energy towards vegetative growth or processes that increase survival rather than towards reproduction (Sletvold and Ågren 2011, 2015a), and thus increase future reproduction by producing more seeds over a longer time period. Short-term demographic effects of pollen limitation have been studied in several species, but very little is known about multiyear responses to reproductive failure caused by pollen limitation (but see Alexandersson and Ågren 1996). This knowledge should be essential to improve our understanding of the lifetime consequences of pollen limitation.

Several factors may contribute to among-population variation in compensation. Most importantly, lifespan should predict compensation. For short-lived species with few reproductive events during their lifetime, the loss of even a single reproductive season could lead to severe fitness reductions, and subsequent increased allocation of resources to flowering can be expected at the expense of allocation to survival. By contrast, for long-lived species with multiple reproductive events, the loss of seed production in a single year may be of minor consequence, and allocation to continued survival may be expected to dominate (Clutton-Brock 1984; Morris and Doak 2004). Even small life history differences between closely related species (e.g., maximum flower production or resource storage capabilities) could potentially impact the ability to increase reproduction to compensate for previous reproductive failures (Sletvold and Ågren 2015b). Moreover, life history differences may affect the length of the compensation period. Short-lived species with limited remaining opportunities for reproduction may allocate a large amount of resources to compensate in the next reproductive effort, whereas long-lived species may compensate by increasing reproduction over multiple ensuing bouts. Comparative studies of species that differ in lifetime pattern of reproduction are thus key to understand allocation to reproduction.

The compensatory ability of the population may also vary with environment. For example, a population experiencing several consecutive years of unfavorable conditions such as low resource availability may be less able to store and allocate resources for increased future reproductive effort (Hawkes and Sullivan 2001). A similar effect is likely for individuals in chronically resource-poor or harsh environments and is supported by studies showing higher costs of reproduction in stressful environments (Sandvik 2001; Obeso 2002; Sletvold and Ågren 2011, 2015a,b). Populations that frequently experience severe pollen limitation are also expected to be less able to compensate than populations that experience pollen limitation only rarely. This is because strong pollen limitation in effect imposes a cap on the level of realized reproduction, regardless of potential compensatory increases in flower production.

We tested the extent of reproductive compensation in two species of long-lived perennial orchids, *Dactylorhiza lapponica* and *Dactylorhiza incarnata subsp. cruenta* (hereafter *D. incarnata*) by experimentally reducing and increasing the pollination rate via flower removal and hand-pollination, respectively, in two annual cohorts and monitoring demographic response in the subsequent 2 years. Both species are deceptive, i.e., they do not produce any reward for their pollinators, and fruit production is strongly pollen limited. No resource limitation of seed production was documented in a previous short-term experiment on *D. lapponica* (Sletvold et al. 2017). *Dactylorhiza incarnata* produces more flowers and fruits per flowering event and has a shorter lifespan compared to *D. lapponica*.

We used our experiment to quantify short-term compensation for loss of reproduction, and to test if the level of compensation depends on life history differences between species, resource status, and year. Specifically, we predicted that (i) individuals are able to compensate for the loss of reproduction in a single year by short-term increases in the probability of reproduction and flower production, or using saved resources to increase survival and growth, (ii) short-term compensation in *D. incarnata* is stronger than in *D. lapponica*, due to a shorter life span, higher flower production and weaker pollen limitation in the former species, (iii) the strength of compensation increases with individual size, due to increased stored resources, (iv) compensation strength varies temporally and this variation may differ between the two species because they differ in responses to climatic variation (cf. Tye et al. 2018).

## Materials and methods

### Study site and populations

The study was conducted at the western side of the Sølendet nature reserve in central Norway (62.68 N, 11.815 E), situated at 710–750 m a.s.l., at the transition between the middle and northern boreal zone. This nature reserve consists of sloping fen habitats that were used for haymaking until the 1950′s, and that now are kept open by extensive mowing (Moen et al. 2012). Climate is characterized by a short growing season, with plants emerging in June and wilting in August. The area holds large populations of both study species, *Dactylorhiza incarnata subsp. cruenta* (L.) Soó and *Dactylorhiza lapponica* (Laest. ex Hartm.) Soó, with a total flowering population size of one to several thousand individuals. Both species occur primarily in fen communities, where *D. incarnata* tends to be found in microsites with higher standing water compared to *D. lapponica.*

The study species are non-clonal, tuberous orchids that produce a leaf rosette that is fully grown by June–July. From late June, flowering individuals produce a single infloresence with purple flowers with no reward. Both species are self-compatible, but require pollinators for successful fruit set. Flowers are pollinated by bumblebees, primarily *Bombus pascuorum* and *B. lucorum*. Fruits mature in August, and a new replacement tuber is formed, which produces a new shoot that emerges next spring. Long-term data at the study site show that *D. incarnata* produces on average 50% more flowers per flowering individual compared to *D. lapponica* (mean ± SD; 11.8 ± 3.7 vs. 7.9 ± 2.1) and has a 61% higher fruit set (proportion of flowers successfully producing fruits, 0.21 ± 0.20 vs. 0.13 ± 0.21). In contrast, *D. lapponica* has a slightly higher average annual survival rate (0.92 ± 0.27 vs. 0.88 ± 0.32) and longer lifespan (A. Moen, D-I. Øien, N. Sletvold; unpublished data). Dormancy rates are typically low (< 5%). The two species also differ in response to variation in climatic factors at the study site. Increasing summer temperature is associated with higher survival and lower dormancy in the following year in *D. lapponica*, but not in *D. incarnata* (Tye et al. 2018).

### Experimental methods

To test compensation ability, we experimentally reduced and increased pollination in a total of four independent reproductive cohorts of individuals: 2014 and 2015 for each of *D. incarnata* and *D. lapponica*. For each cohort, we marked 300 flowering individuals across a total area of approximately 200 × 700 m. Individuals were randomly allocated to one of three treatments; increased pollination by supplemental hand-pollination of all flowers (HP), decreased pollination by removal of all flowers (FR), and natural pollination (open-pollinated controls, C). In this experiment, compensation implies that plants that are pollen limited in the first year (FR and C treatments) sustain a higher fecundity and/or survival and growth in the following 2 years compared to plants with no pollen limitation (HP treatment). After 3 years, an equal fruit production across treatments (HP = C = FR) is consistent with full compensation, HP > C > FR is consistent with undercompensation, and a difference that simply mirrors the initial treatment effect is consistent with no compensation.

Both populations were visited throughout the flowering period, and in the hand-pollination treatment, all flowers were pollinated by hand with cross pollen from the local population and all flowers received supplemental pollination at least once. Pollen was primarily collected from individuals within the hand-pollination treatment. In the flower removal treatment, we removed all flower buds with scissors as they were about to open and noted the total number of flowers removed. We also recorded the size of each plant as total basal leaf area (summed area of the bottom three leaves measured to the nearest mm^2^) by measuring maximum length and width and calculating area under the assumption that the leaves are approximately elliptical in shape. For each cohort, we assessed the number of flowers and fruits produced at the end of the flowering period. In 2016, fruit data from *D. lapponica* were lost, and only flower production was recorded.

In 2015 and 2016, we collected additional demographic data for individuals in cohorts treated in previous years. This information included life history stage [dormant/dead (missing individual, no above-ground biomass), vegetative, flowering], basal leaf area, number of flowers, and number of fruits. Because multiyear vegetative dormancy is possible in both species, we were not able to differentiate dormancy from mortality in this study. To compare total reproductive output among treatments, we calculated pooled flower and fruit production for each individual across all years studied. We quantified pollen limitation (PL) as 1—(mean fruit production of open-pollinated control plants/mean fruit production of hand-pollinated plants). In June 2014, a late frost episode caused a substantial reduction in sample size of *D. lapponica*. In other cohorts, premature wilting or flag displacement caused only minor reductions in sample size (all final sample sizes are given in Table S1).

### Data analysis

We used generalized linear models to examine the effects of pollination treatment (FR, C, HP), species (DIC, DL), cohort (2014, 2015) and their interactions on plant size, number of flowers and fruits in the first year (the year of treatment), and on survival, probability of flowering, plant size and number of flowers in the second year. The flower removal treatment was excluded in the analysis of number of fruits in the first year. In these models, we tested whether compensation differed between species by including the pollination treatment by species interaction. Fruit production was analysed separately by year, because we lacked data for *D. lapponica* in 2016. We also used a generalized linear model to examine the effects of pollination treatment (FR, C, HP) and species (DIC, DL) on plant size and fitness components in the third year (only quantified for the 2014 cohort). Survival (emerging vs. missing) and flowering probability (flowering vs. non-flowering, including only surviving plants) were analysed with binomial errors and a logit link function (proc GENMOD; SAS 9.3, SAS Institute Inc., Cary, NC, USA), while size, and number of flowers and fruits (including only reproductive plants) were analysed with normal errors and identity link (proc GLM). In *D. lapponica*, survival was uniformly high, and survival of *D. incarnata* was also analysed separately. In cases with a significant treatment effect, we performed post hoc comparisons using a Tukey–Kramer correction for multiple testing. To determine whether compensation is size dependent, we also included size and its interaction with treatment in the statistical models. The pollination by size interaction was never statistically significant, and was dropped from final models. Finally, we used a two-way ANOVA to examine the effects of pollination treatment (FR, C, HP), species (DIC, DL), and their interaction on total number of flowers (pooled across years). The two cohorts were analysed separately, because we had 3 years of data for the 2014 cohort and 2 years of data for the 2015 cohort. Total fruit production (pooled across years) was analysed separately by species, because data from 2016 were available only for *D. incarnata*.

## Results

### Size, flower, and fruit production in the first year

The size (basal leaf area) of individuals did not differ between pollination treatment groups at the onset of the experiment for any of the species or cohorts (Table 1, Fig. S1a), and there was no effect of pollination treatment on flower production in the first year (Table 1). Supplemental hand-pollination increased fruit production in all species–cohort combinations, ranging from a 2.4- to a 6.8-fold increase compared to open-pollinated controls (Tables S1, S2; Fig. S1b). The strength of pollen limitation in the year of treatment differed significantly between species and cohorts (Table 1). In 2014, pollen limitation was stronger in *D. cruenta* than in *D. lapponica* (0.66 vs. 0.59, respectively), whereas the opposite was true in 2015 (0.70 vs. 0.85). In both species, pollen limitation was stronger in 2015 than in 2014. Individuals of *D. lapponica* had larger leaf area but produced fewer flowers and fruits than those of *D. incarnata* in both years (Tables 1, S1). Both species were larger and produced more flowers and fruits in 2015 compared to 2014. Flower and fruit production increased with size in both species (Table 1).

**Table 1:** The effect of pollination treatment (flower removal, open-pollinated control, supplemental hand-pollination), species (*Dactylorhiza incarnata*, *D. lapponica*), cohort (2014, 2015) and their interactions on fitness components in the first (year of treatment; size, number of flowers and fruits) and second year (survival, flowering probability, size, number of flowers) analysed with the GLM (size, number of flowers and fruits; *F*-ratio included below) or GENMOD (survival and flowering probability; *χ*^2^ included below) procedure in SAS

	Poll,* df* = 2		Species,* df* = 1		Cohort,* df* = 1		Poll × Sp,* df* = 2		Poll × Co,* df* = 2		Sp × Co,* df* = 1	Poll × Sp × Co,* df* = 2			SizeYr1^a^,* df* = 1	
	*F*/*χ*^2^	*p*	*F*/*χ*^2^	*p*	*F*/*χ*^2^	*p*	*F*/*χ*^2^	*p*	*F*/*χ*^2^	*p*	*F*/*χ*^2^	*p*	*F*/*χ*^2^	*p*	*F*/*χ*^2^	*p*
Size Yr1 (*n* = 1003)	0.06	0.938	25.8	< 0.0001	101.8	< 0.0001	1.22	0.296	0.66	0.518	0.26	0.610	0.37	0.691	–	–
NumFlow Yr1 (*n* = 1003)	0.26	0.772	785	< 0.0001	0.35	0.557	2.60	0.075	0.75	0.472	10.4	0.001	0.28	0.755	298.6	< 0.0001
NumFruits Yr1^b^ (*n* = 653)	449	< 0.0001	376	< 0.0001	20.9	< 0.0001	70.1	< 0.0001	47.0	< 0.0001	33.0	< 0.0001	1.89	0.170	89.7	< 0.0001
Survival Yr2 (*n* = 999)	0.50	0.780	8.12	0.0044	3.59	0.058	1.29	0.525	0.50	0.780	8.13	0.004	1.29	0.524	1.30	0.255
Flow Yr2 (*n* = 806)	0.21	0.902	0.223	0.637	6.30	0.012	7.38	0.025	0.20	0.903	0.22	0.638	7.39	0.025	60.7	< 0.0001
Size Yr2 (*n* = 805)	9.51	< 0.0001	0.90	0.344	24.6	< 0.0001	2.71	0.067	9.51	< 0.0001	0.89	0.346	2.71	0.067	239.6	< 0.0001
NumFlow Yr2 (*n* = 271)	1.05	0.351	8.53	0.004	4.62	0.033	0.73	0.484	0.13	0.876	0.57	0.451	0.53	0.587	51.6	< 0.0001

Initial size (basal leaf area in year 1) was included as a covariate. Sample size (*n*) for each analysis is given in parentheses

^a^Square root transformed

^b^Excluding the flower removal treatment

### Fitness components in the second year

The effect of pollination treatment on fitness components in the second year differed significantly between species and cohorts (Table 1).

#### Survival

**Fig. 1:**
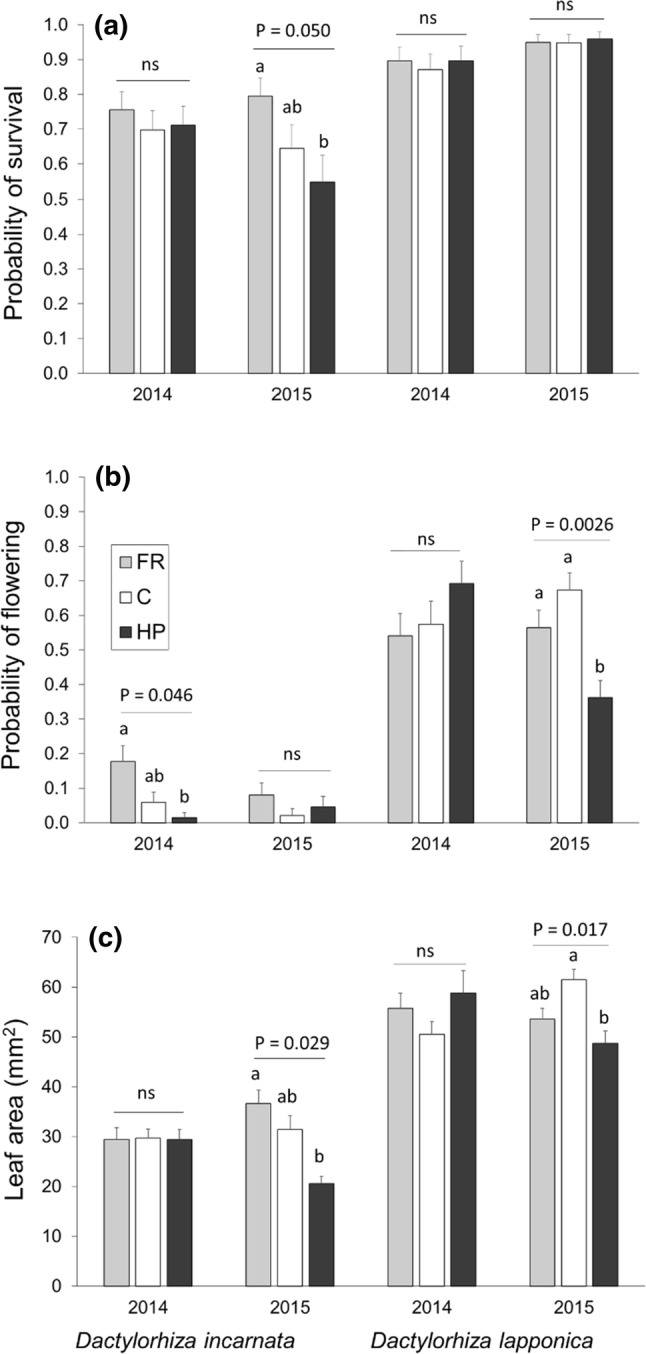
The effect of pollination treatment (*FR* flower removal, *control* natural pollination, *HP* supplemental hand-pollination) in the first year on performance in the second year (mean ± SE) in the 2014 and 2015 cohorts of *Dactylorhiza incarnata ssp. cruenta* and *D. lapponica*: **a** survival, **b** flowering proability, **c** size (basal leaf area in mm^2^). Letters above bars indicate significant differences (*p* < 0.05) between treatment groups identified by posthoc tests (Tukey–Kramer) conducted within the full model using the GLM or GENMOD procedure in SAS. *P* values for all relevant pairwise comparisons are given in Table S2

There was no effect of pollination treatment on survival in the main model (Table 1), reflecting that survival was high and varied little, particularly in *D. lapponica* (Table S1, Fig. 1a). However, when *D. incarnata* was analysed separately, results indicated that hand-pollinated plants had significantly lower survival compared to plants with their flowers removed in 2015 (Tukey *p* = 0.025; poll *χ*^2^ = 7.73, *p* = 0.021, cohort *χ*^2^ = 1.02, *p* = 0.21, poll*coh *χ*^2^ = 3.21, *P* = 0.20; Fig. 1a). In both cohorts, survival was higher in *D. lapponica* than in *D. incarnata* (Table 1).

#### Fecundity

The effect of pollination treatment on the probability of flowering the following year differed between species and cohorts (significant 3-way interaction in Table 1, Fig. 1b, Fig. S2). In the 2014 cohort, hand-pollinated *D. incarnata* plants had significantly lower flowering probability next year than plants with their flowers removed, whereas no effect of pollination was detected in *D. lapponica* (Table S2, Fig. 1b). In the 2015 cohort, hand-pollinated *D. lapponica* plants had significantly lower flowering probability next year than both control and flower removal individuals, whereas no effect of pollination was detected in *D. incarnata* (Table S2, Fig. 1b). In both cohorts, *D. incarnata* had considerably lower flowering probability than *D. lapponica* (Tables 1, S1). For plants that flowered, pollination treatment did not affect number of flowers or fruits produced the second year (Tables 1, Fig. S1c–d). Flowering probability and number of flowers and fruits in the second year increased with size in the first year (Table 1).

#### Size

The effect of pollination treatment on size (basal leaf area) differed between cohorts (Tables 1, Fig. 1c). In the 2014 cohort, pollination treatment did not affect size in any of the two species (Table S2). In the 2015 cohort, hand-pollinated *D. incarnata* individuals were significantly smaller than plants with their flowers removed, and hand-pollinated *D. lapponica* individuals were smaller than control plants (Table S2, Fig. 1c). Individuals of *D. lapponica* were larger than individuals of *D. incarnata* in both cohorts, and size increased with size in the first year (Table 1).

### Fitness components in the third year

There was no statistically significant effect of pollination treatment on survival in the third year in any of the two species (Table 2). Again, survival was higher for *D. lapponica* than for *D. incarnata* (Table S1, Fig. S1e). The effect of pollination treatment on the probability of flowering in the third year tended to differ between species (marginally significant pollination by species interaction in Table 2, *p* = 0.052). In *D. incarnata*, plants in the flower removal treatment had 46% lower probability of flowering compared to control plants, while in *D. lapponica*, the corresponding probability was 23% higher (Table S1, Fig. S1f). There was no effect of pollination treatment on size (Fig. S1g) or number of flowers and fruits (Tables 2, S1). Flowering probability, size, and number of flowers and fruits were all positively related to size in the first year (Table 2).

**Table 2:** The effect of pollination treatment (flower removal, open-pollinated control, supplemental hand-pollination), species (*Dactylorhiza incarnata*, *D. lapponica*), and their interaction on fitness components (survival, flowering probability, size, number of flowers) in the third year analysed with the GLM (size and number of flowers; *F*-ratio included below) or GENMOD (survival and flowering probability; *χ*^2^ included below) procedure in SAS

	Poll,* df* = 2		Species,* df* = 1		Poll × Sp,* df* = 2		SizeYr1,* df* = 1	
	*F*/*χ*^2^	*p*	*F*/*χ*^2^	*p*	*F*/*χ*^2^	*p*	*F*/*χ*^2^	*p*
Survival Yr3 (*n* = 461)	0.031	0.985	21.4	< 0.0001	1.53	0.466	1.60	0.205
Flow Yr3 (*n* = 346)	0.436	0.804	71.0	< 0.0001	5.93	0.052	7.67	0.0056
Size Yr3 (*n* = 325)	0.79	0.456	138.7	< 0.0001	2.02	0.134	55.5	< 0.0001
NumFl Yr3 (*n* = 157)	0.34	0.715	2.94	0.089	1.10	0.334	4.52	0.035

Initial size (basal leaf area in year 1) was included as a covariate. Sample size (*n*) for each analysis is given in parentheses

### Total flower and fruit production

**Fig. 2:**
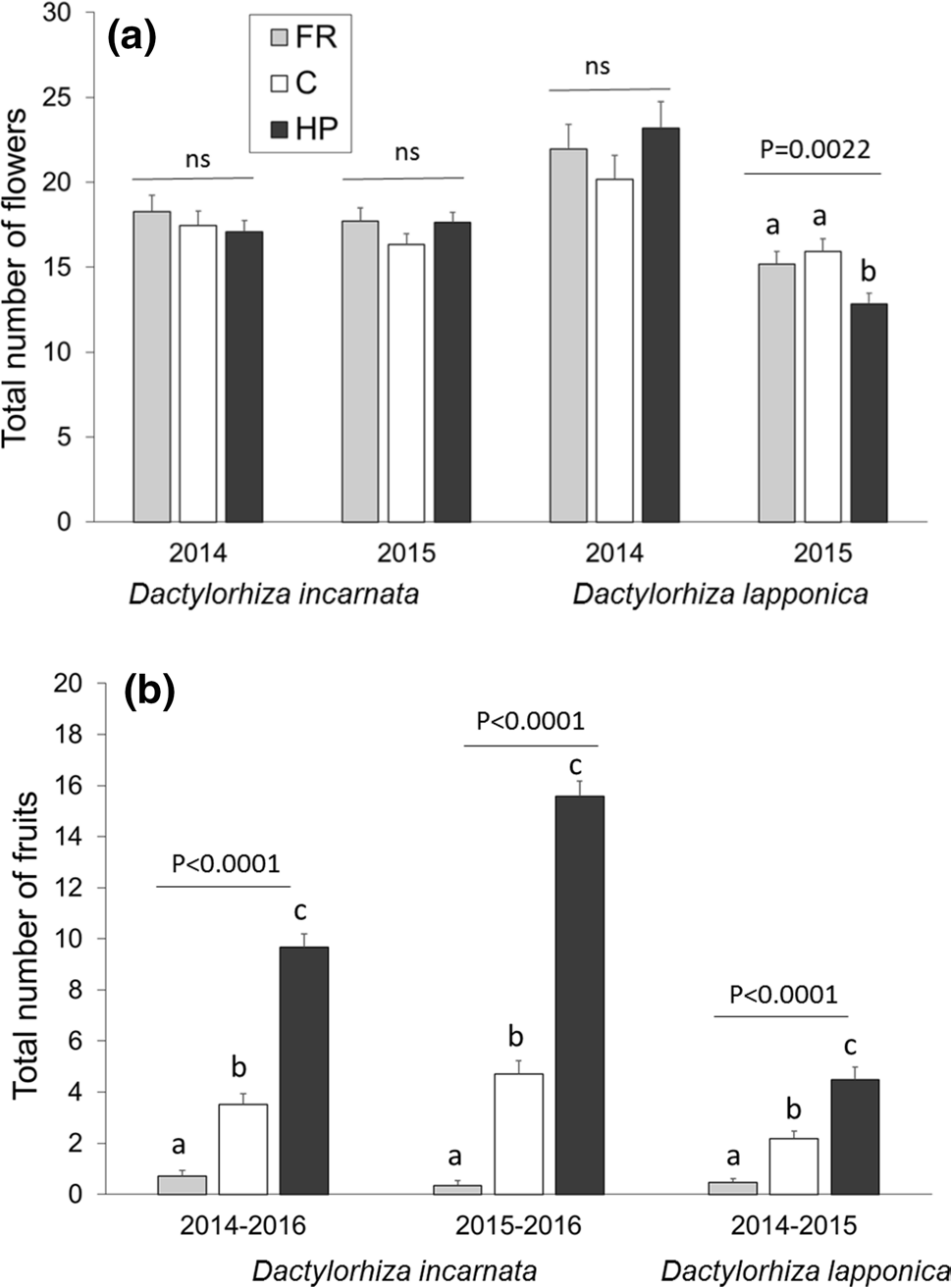
The effect of pollination treatment (*FR *flower removal, *C* control, natural pollination, *HP* supplemetal hand-pollination) in the first year on total number of **a** flowers and **b** fruits produced across study years for the 2014 and 2015 cohorts of *Dactylorhiza incarnata ssp. cruenta* and the 2014 cohort of *D. lapponica*. Statistical significance of the pollination treatment in analyses conducted separately by species and year using the GLM or GENMOD procedure in SAS is indicated above bars. Letters above bars indicate significant differences (*p* < 0.05) between treatment groups identified by post hoc tests (Tukey–Kramer)

Neither of the two species fully compensated for poor pollination by increasing reproduction in the following years. Total flower production was not affected by pollination treatment in the 2014 cohort, whereas the effect differed between species in the 2015 cohort (Table S3). In *D. lapponica*, hand-pollinated plants produced fewer flowers than control (Tukey *p* = 0.0033) and flower removal plants (Tukey *p* = 0.0019), whereas in *D. incarnata*, pollination did not affect total flower production (Tables S1, Fig. 2a). Even though *D. incarnata* produced more flowers per flowering event, the total flower production across 3 years was higher for *D. lapponica*, due to the higher rate of reflowering (2014 cohort in Table S1, Fig. 2a).

Total fruit production was in all cases significantly affected by pollination treatment, and was by far highest in the hand-pollination treatment (Table S1; Fig. 2b). In the 2014 and 2015 cohorts of *D. incarnata*, individuals in the hand-pollination treatment produced 2.8 and 3.3 times as many fruits as individuals in the control treatment, and 13.6 and 47.3 times as many fruits as individuals in the flower removal treatment, respectively (Fig. 2b). In the 2014 cohort of *D. lapponica*, individuals in the hand-pollination treatment produced 2.1 and 9.7 times as many fruits as individuals in the control and flower removal treatment, respectively (Fig. 2b; all Tukey *p *< 0.0001).

## Discussion

In species with multiple reproductive events, reproductive failure in a single year may be compensated by increased future reproductive output or by increased vegetative growth and probability of survival (Obeso 2002; Sletvold and Ågren 2011, 2015a, b). While we observed partial compensation in some species–cohort combinations, demographic effects were generally weak, and all cohorts showed substantially lower reproductive output than would be necessary for full compensation over the 2-year period. In line with our predictions, the species with a shorter life span and higher flower production, *D. incarnata,* tended to show a higher ability to compensate for reduced reproductive output than *D. lapponica*. Overall, the modest short-term increases in demographic rates combined with persistent pollen limitation of seed production make it unlikely that these species can make up for a lost reproductive opportunity.

Both species showed some ability to compensate for an episode of strong pollen limitation. In *D. incarnata*, individuals in the flower removal treatment were larger and had higher flowering probability (2014 cohort) or showed a tendency of a higher survival probability (2015 cohort) compared to individuals in the hand-pollination treatment, while open-pollinated individuals were intermediate of the other treatments (Fig. 1). In *D. lapponica*, individuals in the flower removal and control treatments were larger and had higher flowering probability than hand-pollinated individuals (2015 cohort). However, these demographic effects did not mediate a sufficient increase in fruit production to compensate the initial differences created in the treatment year (Fig. 2). In fact, only *D. incarnata* plants that were prevented from producing any fruits in the first year produced more fruits than other treatments in the two following years (0.7 fruits per individual in FR compared to 0.3 in C and HP). In *D. lapponica*, individuals in all treatments on average produced 0.5 additional fruits in the two following years. As a result, total fruit production of the 2014 cohort of *D. incarnata* and *D. lapponica* across the three study years was 3 and 2 times higher in hand-pollinated plants compared to open-pollinated plants, and 14 and 10 times higher compared to flower removal plants, respectively. This means that it would on average take 15 reproductive events for the flower removal individuals of *D. incarnata* to catch up with the open-pollinated individuals and 46 reproductive events to catch up with the hand-pollinated individuals, given that the documented 0.4 fruit advantage per two years was to persist. This is unlikely, as few individuals flower more than maximum 5–10 times during their lifetime (A. Moen, D-I. Øien, N. Sletvold, M. Tye; unpublished data). Additionally, any compensation in terms of increased demographic rates in the flower removal and control treatments only extended to the next year, with no discernible advantage 2 years after the pollination treatment (Fig. S1). Taken together, the results suggest that pollen limitation in a single year may incur a substantial lowering of lifetime reproductive output, even in such long-lived perennial plants (cf. Burd 1994, 2016).

We did not find any marked difference between species in compensatory ability, although the species with shorter lifespan and higher investment in reproduction, *D. incarnata,* tended to show higher rates of demographic compensation than *D. lapponica*. Somewhat paradoxically, this occurred despite a very low rate of reflowering in *D. incarnata* and was mainly due to a higher flower and fruit production among the few individuals that reproduced compared to *D. lapponica*. The combination of a longer lifespan, higher reflowering rate, and lower pollination success in *D. lapponica* compared to *D. incarnata*, indicate that each individual of *D. lapponica* is likely to spread its lifetime reproductive effort more evenly across more years. This means that a single poor pollination year may have less effect on overall population dynamics in *D. lapponica*.

To what extent the reduced seed production will limit population growth rate depends on the balance between seed number and suitable germination sites (Eriksson and Ehrlén 1992; Tye et al. 2017; Campbell et al. 2017). Addition of pollen in *Lathyrus vernus* (Ehrlén and Eriksson 1995), and of seeds in multiple populations of *Dracocephalum austriacum* (Castro et al. 2015) did not increase population growth rates even though individual seed production was significantly pollen limited in both species, whereas pollen limitation has been shown to constrain population growth in other perennials (Bierzychudek 1982; Maron et al. 2014; Baer and Maron 2018). In the studied orchid populations, sites are kept open by mowing, and the frequency of mowing strongly influences the balance between seed density and recruitment (Sletvold et al. 2010). Mowing opens up gaps and reduces competition, which increases population growth rate through higher recruitment rates (Sletvold et al. 2010). There is no evidence of seed or protocorm survival beyond the first year (Øien et al. 2008), and the lack of a seed bank should cause mowed populations to depend on seed input to maintain population viability, especially since mowing might damage established individuals (Sletvold et al. 2013). It is thus likely that persistent pollen limitation will reduce population viability and future abundance.

The observed lack of reproductive compensation contrasts with the notion that plants that can redistribute resources temporally should not be limited by either pollen or resource availability over longer time intervals (Casper and Niesenbaum 1993; Knight et al. 2005). The driving mechanisms behind this weak compensation for pollen limitation are the relatively low rate of reflowering, a finite limit on flower production within a year, and low pollination success. Flower initiation is controlled by the plant, and efficient pollination may require an ‘over-production’ of flowers, as indicated by the strong pollinator-mediated selection for more flowers documented in several deceptive species, including the *D. lapponica* population studied here (Sletvold et al. 2010; Sletvold and Ågren 2014). Such ‘over-production’ of flowers (i.e. ovules) also allows individuals to capitalize on unusually large pollen loads in a stochastic environment (Burd 1995; Knight et al. 2005). In contrast, fruit set is a function of pollination intensity (Sletvold et al. 2010, 2017) and, therefore, largely determined by external factors. Chronic pollen limitation thus limits the opportunity to compensate for unusually poor pollination years, despite increased allocation to flowering. Somewhat counterintuitively, this suggests that species that currently experience strong pollen limitation may be particularly sensitive to future reductions in pollination, despite that the magnitude of reduction may be less for these species compared to more weakly pollen limited ones.

We also predicted that the ability to compensate should be size dependent (cf. Lawrence 1993; Worley and Harder 1996, Jacquemyn et al. 2010; Miller et al. 2012). However, although size in the year of treatment significantly influenced subsequent performance in the majority of species–cohort combinations (Table 1, Fig. S2), these effects did not lead to stronger compensation by large individuals (no significant pollination treatment by size interactions). In sum, these results are in line with previous studies in orchids that have shown that performance is size dependent, whereas short-term costs of reproduction are not (Sletvold and Ågren 2011, 2015a).

In both species, differences in demographic responses between cohorts were present. In *D. lapponica*, effects of pollination treatment on subsequent performance were observed only in the 2015 cohort. This may be due to considerably stronger pollen limitation compared to the 2014 cohort (0.85 vs. 0.59), which reflected both a lower natural pollination success and a stronger effect of the hand-pollination treatment in 2015 compared to 2014 (Table S1). Stronger pollen limitation in the 2015 cohort may also explain why different fitness components of *D. incarnata* were affected in the 2 years (Fig. 1). Because the fitness of long-lived organisms typically is more sensitive to variation in survival than variation in fecundity (Morris and Doak 2004), demographic responses to changes in reproductive investment are expected to occur via fecundity prior to growth or survival (Sletvold and Ågren 2015a). Although the yearly difference in pollen limitation in *D. incarnata* was modest (0.66 in 2014 vs. 0.70 in 2015), demographic rates can have markedly non-linear responses across this intermediate range of reproductive investment (see Fig. 3 in Sletvold and Ågren 2015b), and the significant effects on survival and size in 2015 versus on fecundity in 2014 are in line with the predicted sequence of fitness components to respond. In addition, environmental variation may have contributed to cohort effects. Models based on 32 years of demographic data including both study species document species-specific associations between variation in climatic factors and demographic rates (Tye et al. 2018), and earlier experiments have documented climate-dependent costs of reproduction in *D. lapponica* (Sletvold and Ågren 2015b).

In conclusion, this study suggests that both *D. lapponica* and *D. incarnata* are unable to compensate effectively for a year of low reproduction due to failure of pollination. The reproductive output and long-term persistence of these populations are thus at risk under scenarios of increasing stochastic variation in pollination rates. Moreover, the differences found between the two species reiterate the importance of considering the effects of even seemingly small differences in life history when predicting the effects of environmental change (cf. Coutts et al. 2016; Che-Castaldo et al. 2018; Tye et al. 2018). The differences between cohorts are also relevant given that many studies of pollen limitation and its consequences are based on a single annual transition. More multiyear studies are needed to fully address the demographic consequences of variation in pollination rates.

## Electronic supplementary material

Below is the link to the electronic supplementary material.

Supplementary file1 (PDF 164 kb)

Supplementary file2 (PDF 148 kb)

Supplementary file3 (DOCX 23 kb)

## References

[CR1] Alexandersson R, Ågren J (1996). Population size, pollinator visitation and fruit production in the deceptive orchid *Calypso bulbosa*. Oecologia.

[CR2] Ashman T-L, Knight TM, Steets JA, Amarasekare P, Burd M, Campbell DR, Mitchell RJ (2004). Pollen limitation of plant reproduction: ecological and evolutionary causes and consequences. Ecology.

[CR3] Baer KC, Maron JL (2018). Pre-dispersal seed predation and pollen limitation constrain population growth across the geographic distribution of *Astragalus utahensis*. J Ecol.

[CR4] Bierzychudek P (1982). The demography of jack-in-the-pulpit, a forest perennial that changes sex. Ecol Monogr.

[CR5] Biesmeijer JC, Roberts SP, Reemer M, Ohlemüller R, Edwards M, Peeters T, Thomas C (2006). Parallel declines in pollinators and insect-pollinated plants in Britain and the Netherlands. Science.

[CR6] Burd M (1994). Bateman’s principle and plant reproduction: the role of pollen limitation in fruit and seed set. Bot Rev.

[CR7] Burd M (1995). Ovule packaging in stochastic pollination and fertilization environments. Evolution.

[CR8] Burd M (2016). Pollen limitation is common—should it be? (A comment on Rosenheim et al., “Parental optimism versus parental pessimism in plants: how common should we expect pollen limitation to be?”. Am Nat.

[CR9] Campbell DR, Brody AK, Price MV, Waser NM, Aldridge G (2017). Is plant fitness proportional to seed set? an experiment and a spatial model. Am Nat.

[CR10] Casper BB, Niesenbaum RA (1993). Pollen versus resource limitation of seed production: a reconsideration. Curr Sci.

[CR11] Castro S, Dostálek T, van der Meer S, Oostermeijer G, Münzbergova Z (2015). Does pollen limitation affect population growth of the endangered *Dracocephalum austriacum* L.?. Popul Ecol.

[CR12] Che-Castaldo J, Che-Castaldo C, Neel MC (2018). Predictability of demographic rates based on phylogeny and biological similarity. Conserv Biol.

[CR13] Clutton-Brock TH (1984). Reproductive effort and terminal investment in iteroparous animals. Am Nat.

[CR14] Coutts SR, Salguero-Gómez R, Csergő AM, Buckley YM (2016). Extrapolating demography with climate, proximity and phylogeny: approach with caution. Ecol Lett.

[CR15] Easterling MR, Ellner SP, Dixon PM (2000). Size-specific sensitivity: applying a new structured population model. Ecology.

[CR16] Ehrlén J, Eriksson O (1995). Pollen limitation and population growth in a herbaceous perennial legume. Ecology.

[CR17] Eriksson O, Ehrlén J (1992). Seed and microsite limitation of recruitment in plant populations. Oecologia.

[CR18] Hawkes CV, Sullivan JJ (2001). The impact of herbivory on plants in different resource conditions: a meta-analysis. Ecology.

[CR19] Jacquemyn H, Brys R, Jongejans E (2010). Size-dependent flowering and costs of reproduction affect population dynamics in a tuberous perennial woodland orchid. J Ecol.

[CR20] Knight T, Steets JA, Vamosi JC, Mazer SJ, Burd M, Campbell DR (2005). Pollen limitation of plant reproduction: pattern and process. Annu Rev Ecol Evol Syst.

[CR21] Larson BMH, Barrett SCH (2000). A comparative analysis of pollen limitation in flowering plants. Biol J Lin Soc.

[CR22] Lawrence WS (1993). Resource and pollen limitation: plant size-dependent reproductive patterns in *Physalis longifolia*. Am Nat.

[CR23] Lennartsson T (2002). Extinction thresholds and disrupted plant-pollinator interactions in fragmented plant populations. Ecology.

[CR24] Maron JL, Baers KC, Angert AL (2014). Disentangling the drivers of context-dependent plant–animal interactions. J Ecol.

[CR25] Miller TEX, Williams JL, Jongejans E, Brys R, Jacquemyn H (2012). Evolutionary demography of iteroparous plants: incorporating non-lethal costs of reproduction into integral projection models. Proc R Soc B.

[CR26] Moen A, Lyngstad A, Øien D (2012). Boreal rich fen vegetation formerly used for haymaking. Nordic J Bot.

[CR27] Morris WF, Doak DF (2004). Buffering of life histories against environmental stochasticity: accounting for a spurious correlation between the variabilities of vital rates and their contributions to fitness. Am Nat.

[CR28] Obeso JR (2002). The costs of reproduction in plants. New Phytol.

[CR29] Øien D-I, O’Neill JP, Whigham DF, McCormick MK (2008). Germination ecology of the boreal-alpine terrestrial orchid *Dactylorhiza lapponica* (Orchidaceae). Annales Botanici Fennici.

[CR30] Potts SG, Biesmeijer JC, Kremen C, Neumann P, Schweiger O, Kunin WE (2010). Global pollinator declines: trends, impacts and drivers. Trends Ecol Evol.

[CR31] Samson DA, Werk KS (1986). Size-dependent effects in the analysis of reproductive effort in plants. Am Nat.

[CR32] Sandvik SM (2001). Somatic and demographic costs under different temperature regimes in the late-flowering alpine perennial herb *Saxifraga stellaris* (Saxifragaceae). Oikos.

[CR33] Schär C, Vidale PL, Lüthi D, Frei C, Häberli C, Liniger MA, Appenzeller C (2004). The role of increasing temperature variability in European summer heatwaves. Nature.

[CR34] Sletvold N, Ågren J (2011). Among-population variation in costs of reproduction in the long-lived orchid *Gymnadenia conopsea*: an experimental study. Oecologia.

[CR35] Sletvold N, Ågren J (2014). There is more to pollinator-mediated selection than pollen limitation. Evolution.

[CR36] Sletvold N, Ågren J (2015). Nonlinear costs of reproduction in a long-lived plant. J Ecol.

[CR37] Sletvold N, Ågren J (2015). Climate-dependent costs of reproduction: survival and fecundity costs decline with length of the growing season and summer temperature. Ecol Lett.

[CR38] Sletvold N, Grindeland JM, Ågren J (2010). Pollinator-mediated selection on floral display, spur length and flowering phenology in the deceptive orchid *Dactylorhiza lapponica*. New Phytol.

[CR39] Sletvold N, Tye M, Ågren J (2017). Resource-and pollinator-mediated selection on floral traits. Funct Ecol.

[CR40] Tye MR, Ferrer-Cervantes ME, Sánchez AM, García-Cervigón AI, Escudero A, Albert MJ, Quintana-Ascencio PF (2017). Assessing seed and microsite limitation on population dynamics of a gypsophyte through experimental soil crust disturbance and seed addition. Plant Ecol.

[CR41] Tye M, Dahlgren JP, Øien D-I, Moen A, Sletvold N (2018). Demographic responses of orchids to climate variation depend on life history and local habitat. Biol Cons.

[CR42] Worley AC, Harder LD (1996). Size-dependent resource allocation and costs of reproduction in *Pinguicula vulgaris* (Lentibulariaceae). J Ecol.

[CR43] Sletvold N, Dahlgren J, Øien D-I, Moen A, Ehrlén JP (2013). The effect of land use practice on the viability of a rare orchid depends on climatic conditions: a 30-year experimental study. Glob Change Biol.

