# Supplementary figures and images for: Pollen limitation in a single year is not compensated by future reproduction
Source: Oecologia. 2020 Feb 20;192(4):989–97. doi: 10.1007/s00442-020-04623-x (PMC7165156; doi:10.1007/s00442-020-04623-x)

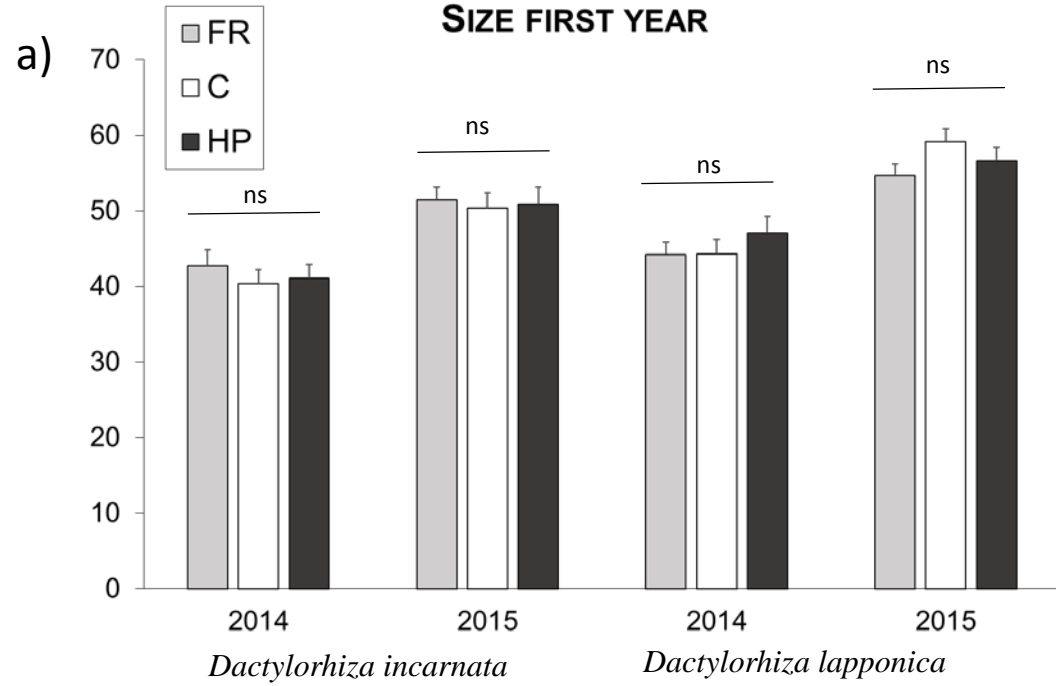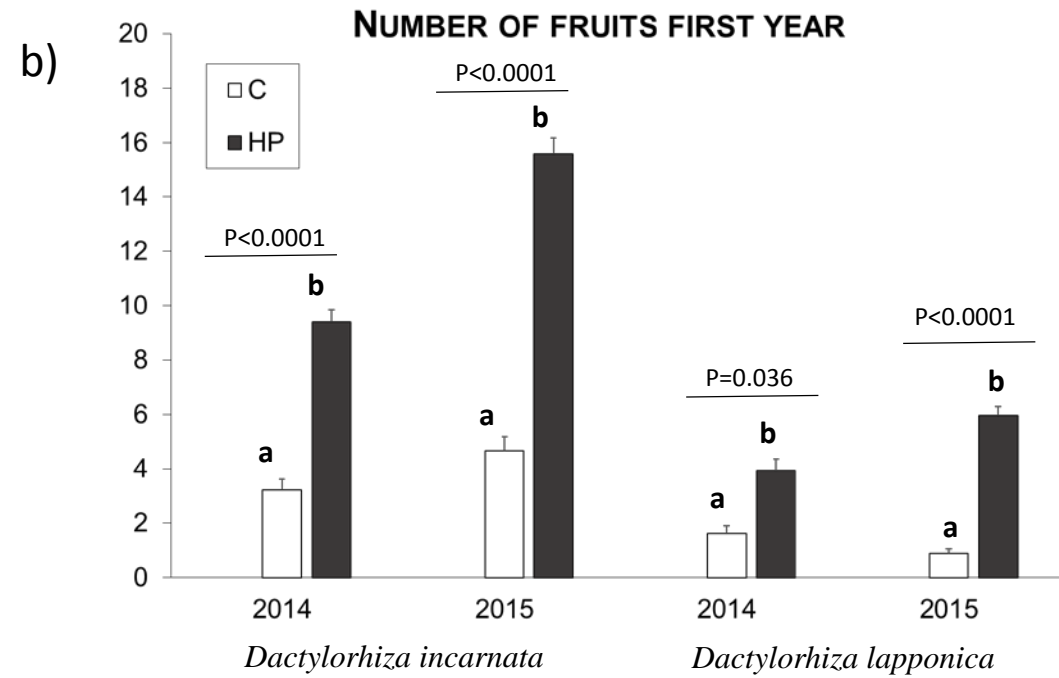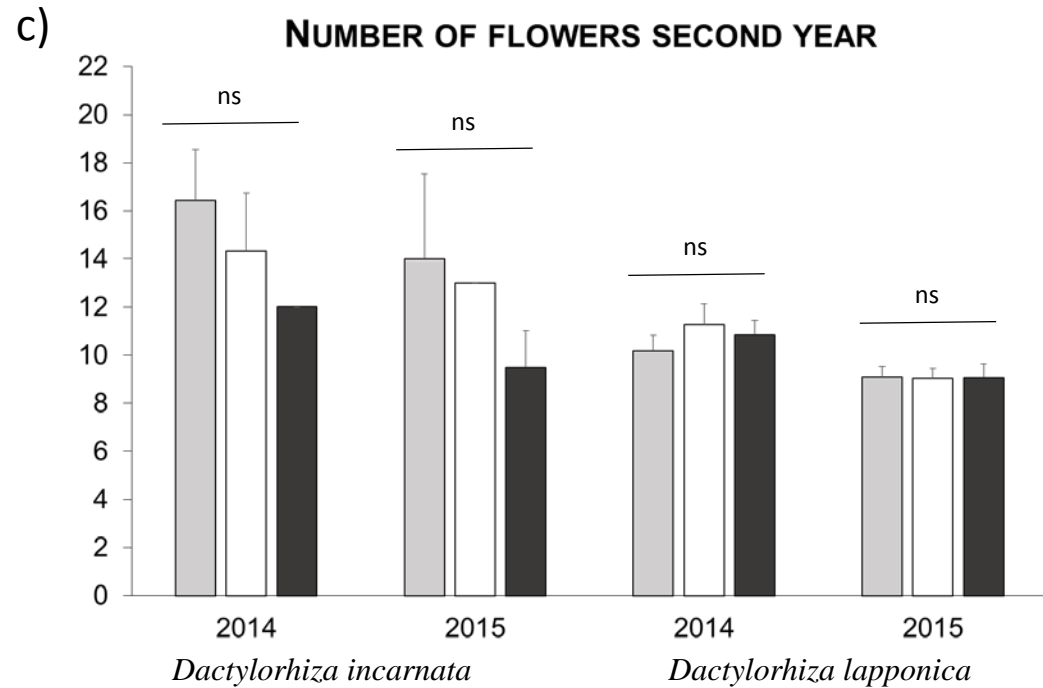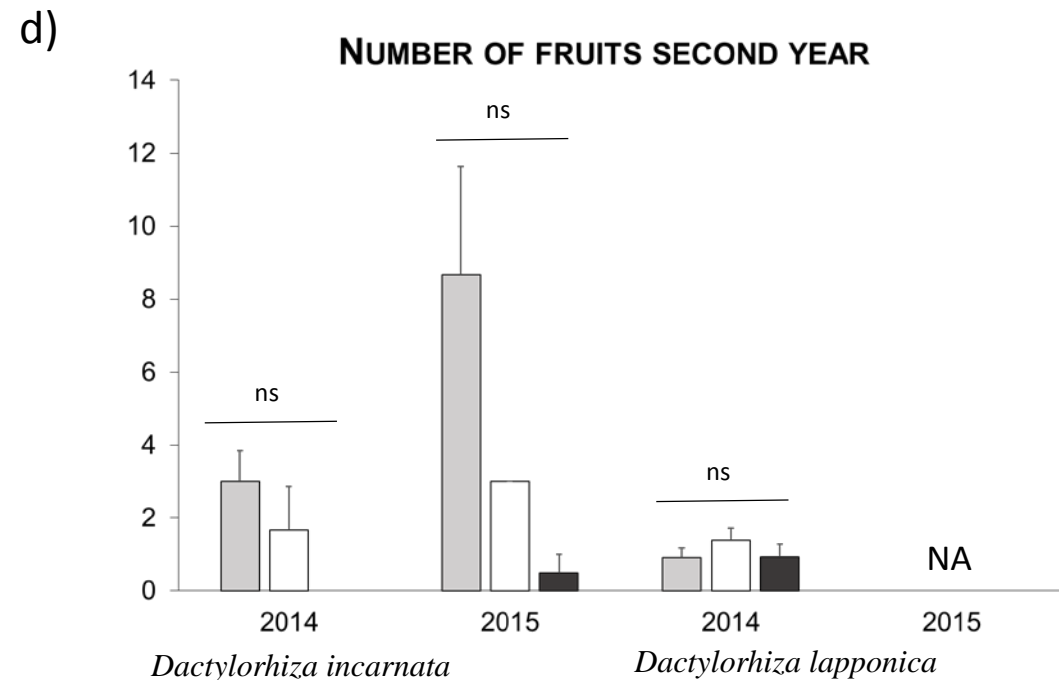

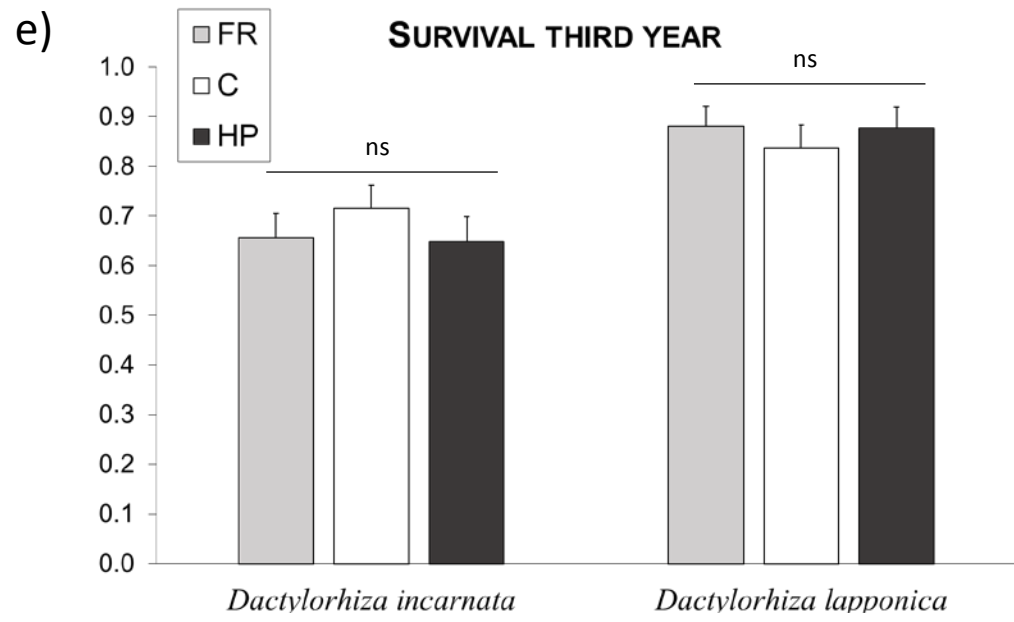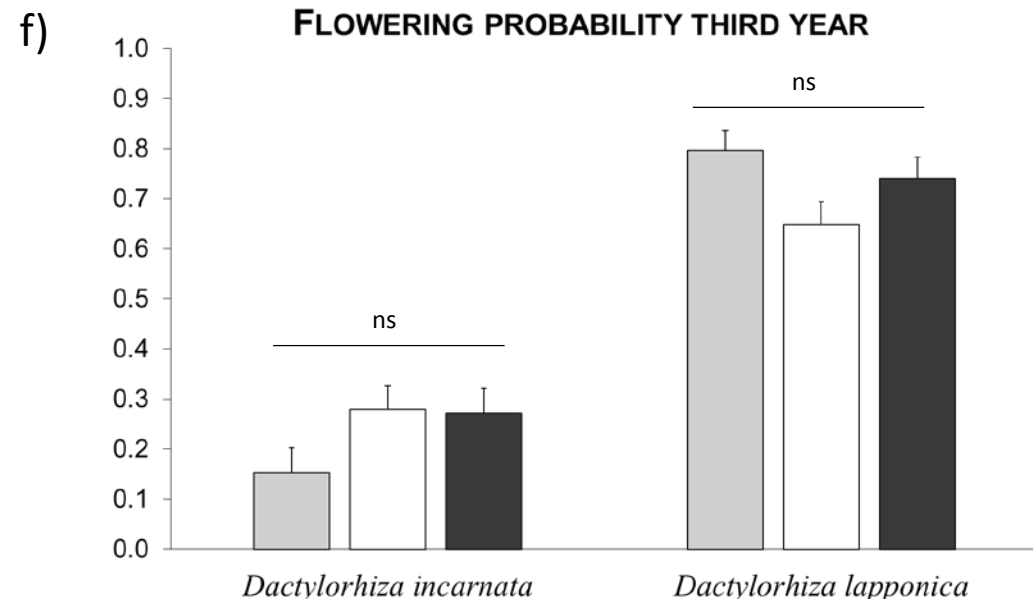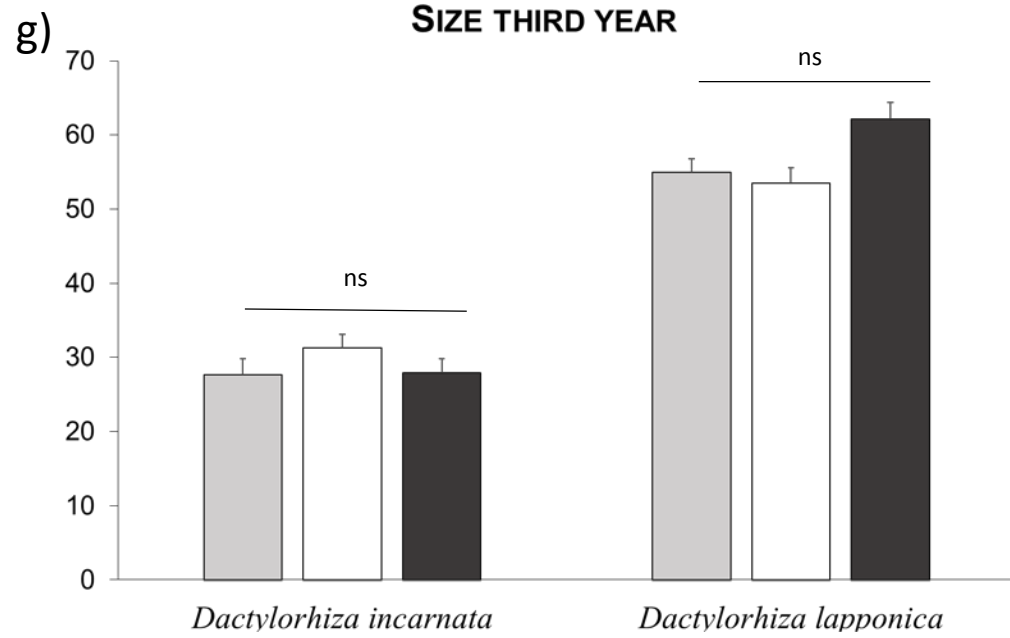

Supplement: Supplementary file 1 — Supplementary file1 (PDF 164 kb) [file 442_2020_4623_MOESM1_ESM.pdf]

a) 2014

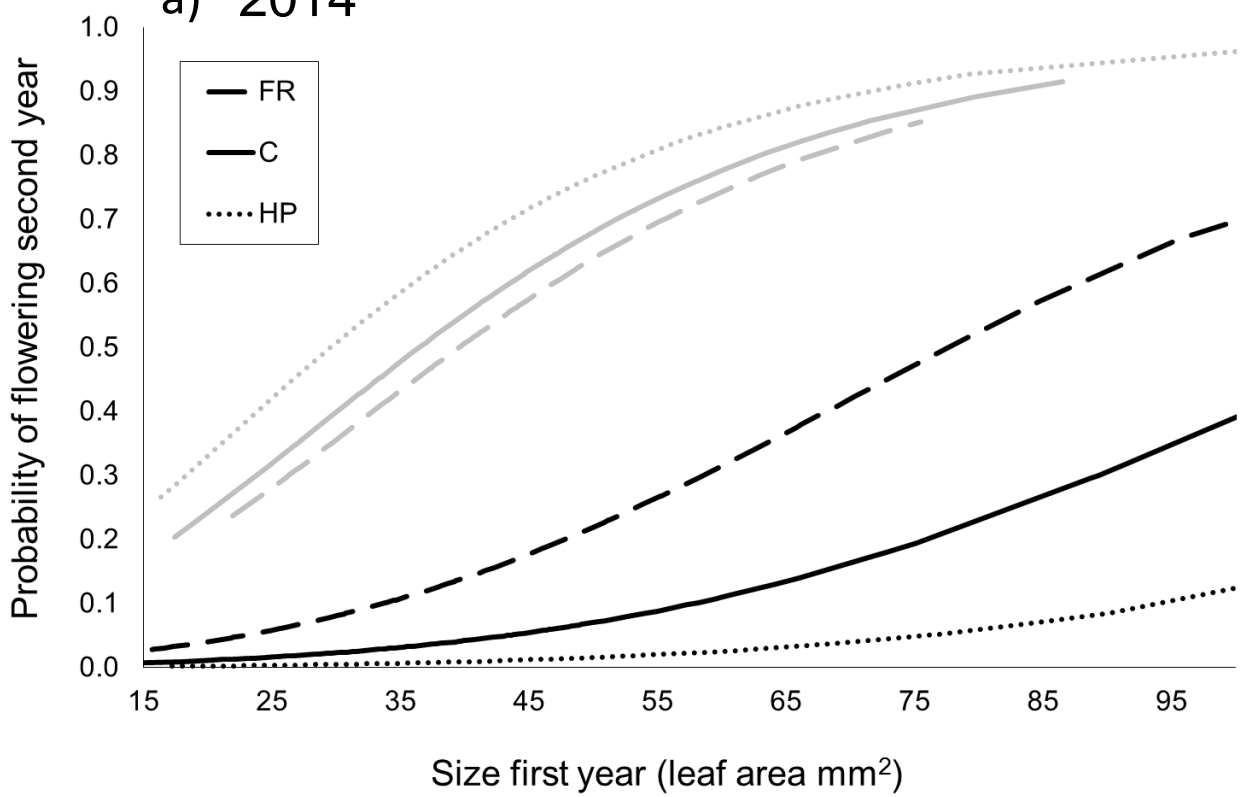

b)

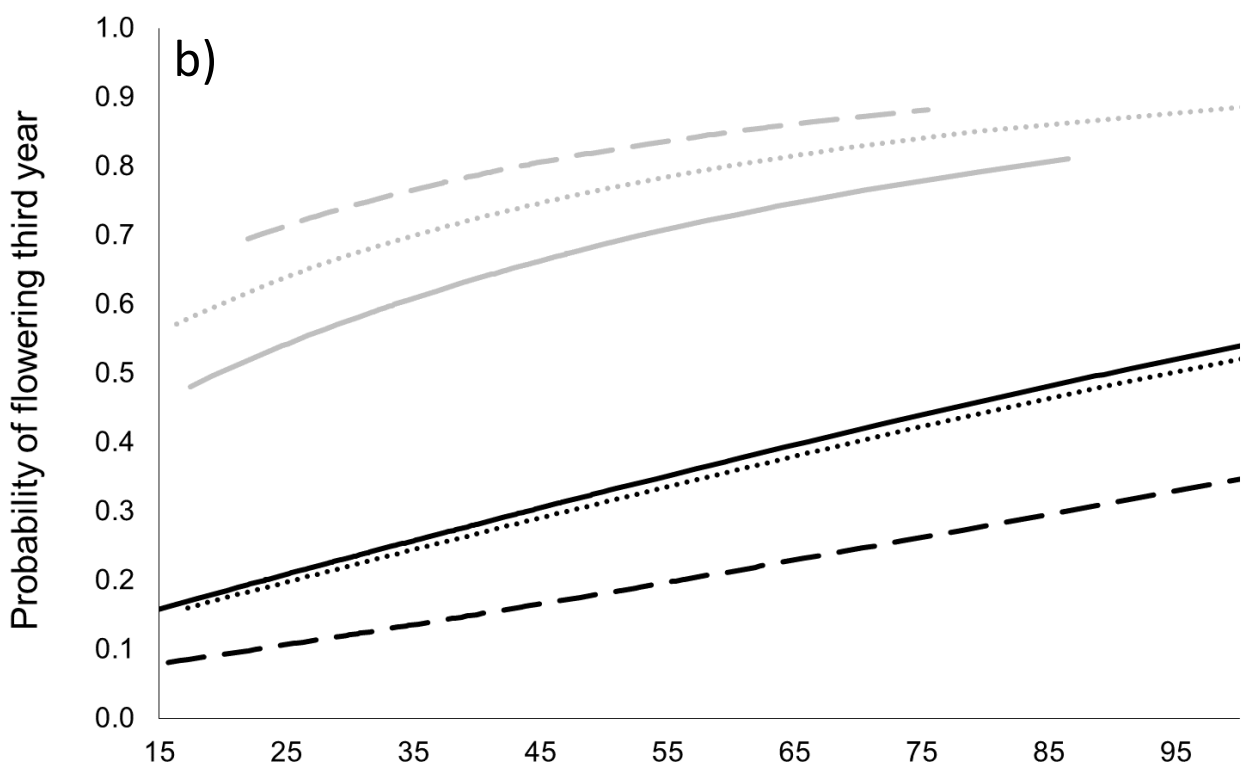

c) 2015

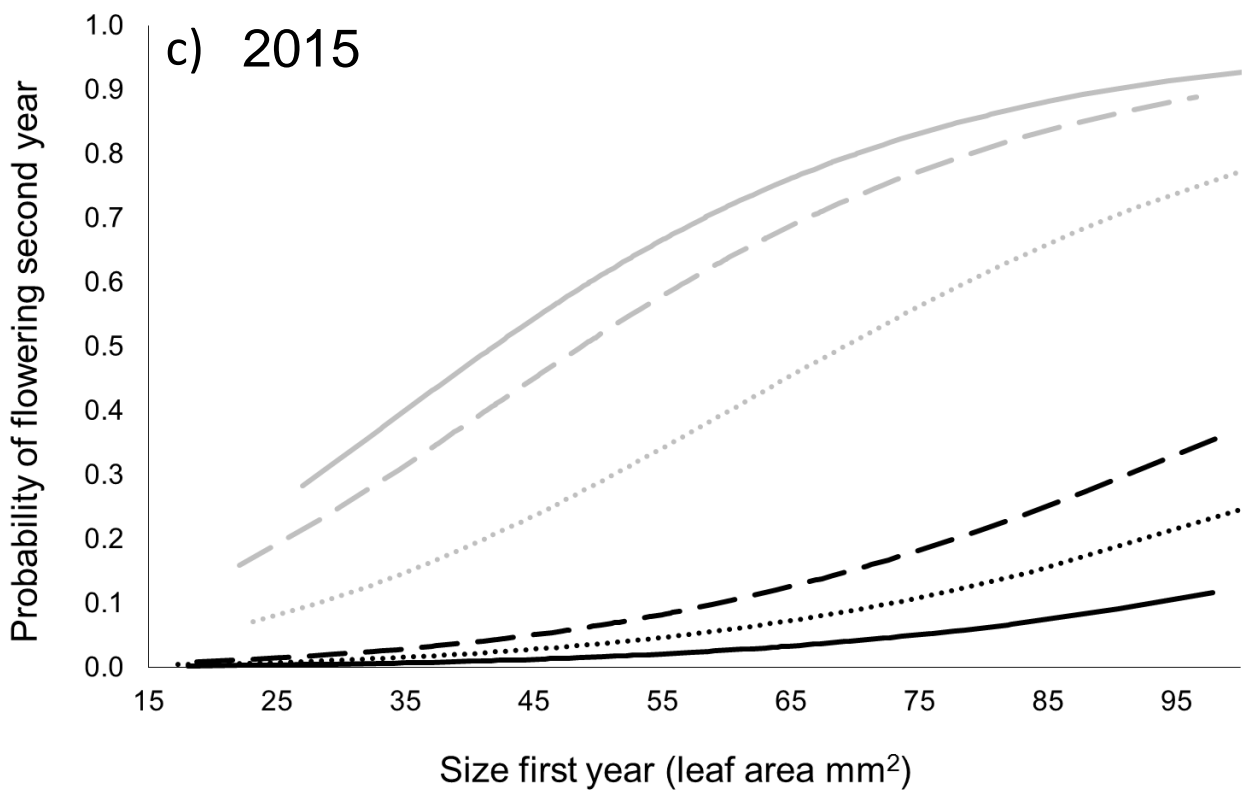

Supplement: Supplementary file 2 — Supplementary file2 (PDF 148 kb) [file 442_2020_4623_MOESM2_ESM.pdf]
