# Supplementary material for: Pollen limitation in a single year is not compensated by future reproduction
Source: Oecologia. 2020 Feb 20;192(4):989–97. doi: 10.1007/s00442-020-04623-x (PMC7165156; doi:10.1007/s00442-020-04623-x)
Supplement: Supplementary file 3 — Supplementary file3 (DOCX 23 kb) [file 442_2020_4623_MOESM3_ESM.docx]

**Supporting information for: Pollen limitation in a single year is not compensated by future reproduction**

**Matthew Tye^1^, Johan P. Dahlgren^2^, and Nina Sletvold^1*^**

**Table S1.** Descriptive statistics (mean ±SD) and sample size for all fitness components.

**Table S2.** Effects of pollination treatment on fitness components in the first and second year as indicated by Tukey-Kramer posthoc tests.

**Table S3.** The effect of pollination treatment and species on total flower production.

**Table S4.** The effect of pollination treatment on total fruit production.

**Figure S1.** Fitness components (mean ±SE) in the first, second and third year.

**Figure S2.** Flowering probability in the second and third year in relation to pollination treatment and size in the first year.

**Tables**

Table S1. Sample size (n) and fitness components (mean±SD) in the first (size, number of flowers and fruits Yr1), second (survival, flowering probability, size, number of flowers and fruits Yr2) and third year (survival, flowering probability, size, number of flowers and fruits Yr3) for three pollination treatments (FR= flower removal, C =open-pollinated control, HP= supplemental hand-pollination) in two cohorts (2014 and 2015) each of *Dactylorhiza incarnata* and *D. lapponica*. Total number of flowers and fruits (summed across study years) are also given. Size is quantified as basal leaf area in mm^2^.

|  | ***Dactylorhiza incarnata ssp. cruenta*** | | | | | | ***Dactylorhiza lapponica*** | | | | | |
| --- | --- | --- | --- | --- | --- | --- | --- | --- | --- | --- | --- | --- |
|  | **2014** | | | **2015** | | | **2014** | | | **2015** | | |
|  | **FR** | **C** | **HP** | **FR** | **C** | **HP** | **FR** | **C** | **HP** | **FR** | **C** | **HP** |
| n | 90 | 96 | 97 | 79 | 77 | 82 | 68 | 62 | 58 | 99 | 97 | 98 |
| Size Yr1 | 42.7±19.8 | 40.4±18.1 | 41.0±18.0 | 51.4±15.7 | 50.3±17.7 | 50.9±20.3 | 44.2±13.1 | 44.3±14.5 | 47.1±16.2 | 54.6±14.8 | 59.1±17.5 | 56.6±17.4 |
| n | 90 | 96 | 97 | 79 | 77 | 82 | 68 | 62 | 58 | 99 | 97 | 98 |
| NumFl Yr1 | 14.8±4.6 | 14.9±4.9 | 15.2±4.8 | 16.8±4.7 | 16.2±5.5 | 17.4±5.0 | 9.7±3.3 | 9.6±3.1 | 9.6±2.6 | 10.3±3.5 | 10.3±3.4 | 9.7±3.1 |
| n | 90 | 96 | 97 | 79 | 72 | 82 | 68 | 58 | 57 | 99 | 97 | 94 |
| NumFr Yr1 | 0.00±0.00 | 3.22±3.87 | 9.39±4.63 | 0.00±0.00 | 4.67±4.38 | 15.6±5.54 | 0.00±0.00 | 1.62±2.16 | 3.93±3.27 | 0.00±0.00 | 0.88±1.58 | 5.95±3.18 |
| n | 90 | 96 | 97 | 78 | 76 | 80 | 68 | 62 | 58 | 99 | 97 | 98 |
| Survival Yr2 | 0.76±0.43 | 0.70±0.46 | 0.71±0.46 | 0.79±0.41 | 0.64±0.48 | 0.55±0.50 | 0.90±0.31 | 0.87±0.34 | 0.90±0.31 | 0.95±0.22 | 0.95±0.22 | 0.96±0.20 |
| n | 68 | 67 | 69 | 62 | 49 | 44 | 61 | 54 | 52 | 94 | 92 | 94 |
| Flow Yr2 | 0.18±0.38 | 0.06±0.24 | 0.01±0.12 | 0.08±0.27 | 0.02±0.14 | 0.05±0.21 | 0.54±0.50 | 0.57±0.50 | 0.69±0.47 | 0.56±0.50 | 0.67±0.47 | 0.36±0.48 |
| n | 68 | 67 | 69 | 62 | 49 | 44 | 61 | 54 | 52 | 94 | 92 | 94 |
| Size Yr2 | 29.4±19.2 | 29.7±14.6 | 29.4±17.0 | 36.6±21.2 | 31.4±19.1 | 20.5±10.2 | 55.8±23.5 | 50.5±18.6 | 58.8±32.7 | 53.6±20.7 | 61.5±20.3 | 48.7±23.9 |
| n | 12 | 3 | 1 | 5 | 1 | 2 | 33 | 30 | 36 | 53 | 61 | 34 |
| NumFl Yr2 | 16.4±7.3 | 14.3±4.2 | 12.0±0.0 | 14.0±7.9 | 13.0±0.0 | 9.5±2.1 | 10.2±3.7 | 11.3±4.7 | 10.8±3.7 | 9.1±3.3 | 9.0±3.4 | 9.1±3.5 |
| n | 12 | 3 | 1 | 3 | 1 | 2 | 32 | 26 | 33 |  |  |  |
| NumFr Yr2 | 3.00±2.92 | 1.67±2.08 | 0.00±0.00 | 8.67±5.13 | 3.00±0.00 | 0.50±0.71 | 0.91±1.55 | 1.38±1.72 | 0.94±1.94 | NA | NA | NA |
| n | 90 | 95 | 91 |  |  |  | 67 | 61 | 57 |  |  |  |
| Survival Yr3 | 0.66±0.48 | 0.72±0.45 | 0.65±0.48 | NA | NA | NA | 0.88±0.33 | 0.84±0.37 | 0.88±0.33 | NA | NA | NA |
| n | 59 | 68 | 59 |  |  |  | 59 | 51 | 50 |  |  |  |
| Flow Yr3 | 0.15±0.36 | 0.28±0.45 | 0.27±0.45 | NA | NA | NA | 0.80±0.41 | 0.65±0.48 | 0.74±0.44 | NA | NA | NA |
| n | 55 | 61 | 50 |  |  |  | 59 | 50 | 50 |  |  |  |
| Size Yr3 | 27.7±18.9 | 31.3±15.6 | 27.9±17.4 | NA | NA | NA | 55.0±24.5 | 53.5±24.9 | 62.1±22.9 | NA | NA | NA |
| n | 9 | 17 | 16 |  |  |  | 47 | 32 | 36 |  |  |  |
| NumFl Yr3 | 12.4±3.32 | 12.0±2.89 | 10.7±2.18 | NA | NA | NA | 10.6±4.83 | 9.8±3.88 | 11.1±3.93 | NA | NA | NA |
|  | 6 | 13 | 12 |  |  |  |  |  |  |  |  |  |
| NumFr Yr3 | 4.67±3.72 | 1.77±1.92 | 2.33±3.87 | NA | NA | NA | NA | NA | NA | NA | NA | NA |
| n | 90 | 96 | 97 | 79 | 77 | 82 | 68 | 62 | 58 | 99 | 97 | 98 |
| TotFlowers | 18.3±9.4 | 17.4±8.4 | 17.1±6.7 | 17.7±6.8 | 16.4±5.5 | 17.6±5.6 | 21.9±12.0 | 20.2±11.1 | 23.2±11.9 | 15.2±7.2 | 15.9±7.2 | 12.8±6.2 |
| n | 90 | 96 | 97 | 79 | 72 | 82 | 68 | 60 | 57 | 99 | 97 | 98 |
| TotFruits | 0.71±2.00 | 3.51±4.17 | 9.68±5.04 | 0.33±1.86 | 4.71±4.35 | 15.6±5.56 | 0.46±1.15 | 2.17±2.38 | 4.47±3.83 | 0.00±0.00 | 0.88±1.58 | 5.95±3.18 |

**Table S2.** P-values from Tukey Kramer tests of pairwise comparisons of the effect of the three pollination treatments (flower removal FR, open-pollinated control C, supplemental hand-pollination HP) on number of fruits in the first year (year of treatment) and on fitness components in the second year (flowering probability and size) in the 2014 and 2015 cohorts of *Dactylorhiza incarnata ssp. cruenta* and *D. lapponica* analysed with the GLM or GENMOD procedure in SAS. Models included pollination treatment, species, cohort and all interactions as fixed factors, as well as initial size (basal leaf area in year 1) as a covariate. Main model results are reported in Table 1.

|  | ***Dactylorhiza incarnata ssp. cruenta*** | | | | | | ***Dactylorhiza lapponica*** | | | | | |
| --- | --- | --- | --- | --- | --- | --- | --- | --- | --- | --- | --- | --- |
|  | **2014** | | | **2015** | | | **2014** | | | **2015** | | |
|  | FR-C | C-  HP | FR-HP | FR-C | C-  HP | FR-HP | FR-C | C-HP | FR-HP | FR-C | C-  HP | FR-HP |
| **NumFr Yr1*** | - | <0.0001 | - | - | <0.0001 | - | - | 0.036 | - | - | <0.0001 | - |
| **FlowYr2** | 0.16 | 0.37 | 0.046 | 0.48 | 0.89 | 0.77 | 0.91 | 0.68 | 0.66 | 0.46 | 0.0026 | 0.071 |
| **SizeYr2** | 0.99 | 0.99 | 0.96 | 0.69 | 0.045 | 0.0029 | 0.56 | 0.37 | 0.69 | 0.18 | 0.0017 | 0.34 |

**Excluding the flower removal treatment*

**Table S3.** The effect of pollination treatment (flower removal FR, open-pollinated control C, supplemental hand-pollination HP), species (*Dactylorhiza incarnata ssp. cruenta*, *D. lapponica*), and their interaction on total flower production (summed across years) in the 2014 and 2015 cohorts analysed with the GLM procedure in SAS. Initial size (basal leaf area in year 1) was included as a covariate.

|  | **Poll, df=2** | | **Species, df=1** | | **Poll×Sp, df=2** | | **SizeYr1^$^, df=1** | |  |
| --- | --- | --- | --- | --- | --- | --- | --- | --- | --- |
| **Total Flowers** | F/χ^2^ | P | F/χ^2^ | P | F/χ^2^ | P | F/χ^2^ | P |  |
| **2014 (n = 471)** | 0.61 | 0.544 | 13.0 | 0.0003 | 1.05 | 0.351 | 104.6 | <0.0001 |  |
| **2015 (n = 532)** | 2.20 | 0.112 | 52.8 | <0.0001 | 5.20 | 0.0058 | 145.2 | <0.0001 |  |

*^$^Square root transformed*

**Table S4.** The effect of pollination treatment (flower removal, open-pollinated control, supplemental hand-pollination) on total number of fruits (summed across years) in the 2014 and 2015 cohorts of *Dactylorhiza incarnata* and *D. lapponica* analysed with the GLM procedure in SAS. Initial size (basal leaf area in year 1) was included as a covariate.

|  | **Poll, df=2** | | **Size Yr1^$^, df=1** | |
| --- | --- | --- | --- | --- |
| **Total Fruits** | F/χ^2^ | P | F/χ^2^ | P |
| *Dactylorhiza incarnata* 2014 (n = 281) | 153.6 | <0.0001 | 59.0 | <0.0001 |
| *Dactylorhiza incarnata* 2015 (n = 233) | 320.9 | <0.0001 | 35.7 | <0.0001 |
| *Dactylorhiza lapponica* 2014 (n = 185) | 36.2 | <0.0001 | 5.92 | 0.0159 |
| *Dactylorhiza lapponica* 2015 (n = 290) | 250.1 | <0.0001 | 14.5 | 0.0002 |

*^$^Square root transformed,*

**Figure legends**

**Figure S1.** The effect of pollination treatment (FR = flower removal, Control = natural pollination, HP = supplemental hand-pollination) in the first year on (a) size and (b) fruit production in the first year, on (c) flower and (d) fruit production in the second year, and on (e) survival, (f) flowering probability and (g) size in the third year (mean ± SE) in the 2014 and 2015 cohorts of *Dactylorhiza incarnata ssp. cruenta* and *D. lapponica*. Statistical significance of the pollination treatment in analyses conducted separately by species and year using the GLM or GENMOD procedure in SAS is indicated above bars. Letters above bars indicate significant differences (P<0.05) between treatment groups identified by posthoc tests (Tukey-Kramer) from the full model.

**Figure S2.** Predicted flowering probability in relation to pollination treatment (FR = flower removal, Control = natural pollination, HP = supplemental hand-pollination) and size (basal leaf area in mm^2^) in the first year for the 2014 and 2015 cohorts of *Dactylorhiza incarnata ssp. cruenta* and *D. lapponica*: a) Probability of flowering in the second year for the 2014 cohorts, b) Probability of flowering in the third year for the 2014 cohorts, c) Probability of flowering in the second year for the 2015 cohorts. Corresponding statistical tests are given in Table 1 and Table 2.
